# Chemotherapy-induced polyneuropathy: diagnostic challenges and the potential of neurofilament as a biomarker for sensory disorders: the CONKO 023-ChemTox Trial

**DOI:** 10.1007/s00415-025-13463-9

**Published:** 2025-10-22

**Authors:** Lars Uwe Stephan, Janine Abel, Wolfgang Boehmerle, Sebastian Schröder, Soo Ann Yap, Nigel Dross Engelbert Schaeper, Sebastian Stintzing, Tobias Bleumer, Uwe Pelzer

**Affiliations:** 1 Department of Hematology, Oncology and Cancer Immunology, Charité-Universitätsmedizin Berlin, Freie Universität Berlin, Humboldt-Universität Zu Berlin, and Berlin Institute of Health, 10117 Berlin, Germany; 2 Department of Internal Medicine, Bundeswehrkrankenhaus Berlin, 10115 Berlin, Germany; 3 Department of Neurology and Experimental Neurology, Charité-Universitätsmedizin Berlin, Freie Universität Berlin, Humboldt-Universität Zu Berlin, and Berlin Institute of Health, 10117 Berlin, Germany; 4 Department of Internal Medicine 1, Hospital of Wolfsburg, Medical Center of the University of Göttingen, 38440 Wolfsburg, Germany; 5 Department of Hematology, Oncology and Palliative Care, Sana Klinikum Lichtenberg, 10365 Berlin, Germany

**Keywords:** Chemotherapy, Neuropathy, Neurofilament light chain, Quality of life

## Abstract

Chemotherapy-induced polyneuropathy (CIPN) is a prevalent side effect impairing patients’ quality of life, often manifesting as dysesthesia or pain in extremities. To minimize therapy-related side effects as well as to improve quality of life, modern approaches grow increasingly more relevant. We conducted a longitudinal, prospective study to examine patients with lymphoma, leukemia, or gastrointestinal malignancies receiving continuous and temporary treatment up to five times over a 12- to 24-month period, beginning prior to chemotherapy. Assessments included comprehensive questionnaires, clinical neurological and electroneurographic examinations and measurements of neurofilament light chain (NFL). One hundred eight patients were enrolled with fifty-six patients having undergone all examinations both before and after chemotherapy. Data showed a significant rise of the mean Total Neuropathy Score (TNSr) after chemotherapy. Results revealed that approximately 75% of patients experienced CIPN. Continuous chemotherapy was associated with a steady increase in TNSr. Older patients, often with pre-existing and underdiagnosed neuropathy, were more frequently and more severely affected. According to medical records, where diagnostics were primarily based on patients’ subjective information, even moderate cases were frequently underdiagnosed. A moderate correlation was observed between the increase in NfL and changes in TNSr (*r* = 0.385, *p* = 0.004). Patients with cancer affecting the central nervous system (CNS) showed elevated baseline NFL levels. In contrast to non-responders, NFL decreased in patients who responded well to therapy. In conclusion, data underline the necessity of improved diagnostic accuracy of CIPN and support the potential of NFL as a biomarker for its detection. Further investigations should assess its potential in daily oncological routine.

*Trial registration number: *EA2/167/21 (date of registration: 12.08.2021).

## Background

Globally, the incidence of hematologic and oncological diseases is still rising [1]. In 2040, the annual number of patients receiving chemotherapy for cancer is projected to reach 15 million [2]. To make matters more difficult, most oncological patients are outpatients and there is a wide variation in oncological therapies used, reflecting the diversity of these diseases and ongoing advances in medicine [3]. Looking at data from the last 50 years, most cancer entities show significantly increased survival rates and better prognosis today [4]. As a consequence of improved management of short-term side effects of chemotherapy, such as nausea and vomiting, and better prognosis of oncological diseases, long-term side effects and quality of life have grown to be more important [5].

A common long-term side effect is chemotherapy-induced polyneuropathy (CIPN) which significantly influences the patients’ quality of life. Its prevalence is reported to range between 30 and 40%, with considerable variation depending on the specific chemotherapeutic agent and cumulative dose [6, 7]. The clinical manifestations of CIPN vary greatly. Frequently reported symptoms include paresthesia, altered temperature or vibration sensation, and neuropathic pain in the distal extremities [8]. Damage to large nerve fibers is associated with impaired vibration sense and proprioception, while damage to small fibers is linked to altered temperature perception and neuropathic pain [9]. Severe nerve fiber damage may also lead to motor deficits, although motor impairment usually follows prior sensory dysfunction [10]. These symptoms—including balance disorders, restless legs, burning pain, and reduced fine motor skills—not only significantly affect patients’ quality of life but may also necessitate chemotherapy dose reductions [11].

Unfortunately, treatment options for CIPN remain limited and often ineffective [12]. Early diagnosis is, therefore, crucial to prevent CIPN, for example by adjusting or reducing neurotoxic agents. For this purpose, continuous follow-ups would be necessary to reassess and adjust therapies in routine clinical practice. This is aggravated by the fact that most oncological patients are treated in outpatient settings, resulting in limited access to advanced neurological diagnostics and a general lack of time. For these reasons, no universally accepted and satisfactory diagnostic standard has been established to date [13]. Alongside symptom questionnaires, clinical neurological examinations are a frequently used, widely available method in routine care. This allows for early detection of signs such as reduced vibration sensation in the extremities [14]. However, limitations include low specificity and a high degree of subjectivity. In contrast, electroneurography (ENG) is more objective, assessing nerve conduction velocity as well as motor and sensory responses to electrical stimuli [15]. However, mainly ENG but also clinical neurological examinations are time-consuming. In addition, ENG requires specialized equipment. Both factors limit its use in oncological routine settings. Therefore, there is a strong clinical need to improve both diagnosis and treatment of CIPN.

One possible approach is the use of biomarkers. Elevated levels of neurofilament light chain (NFL) in serum or cerebrospinal fluid, a biomarker used in neurodegenerative disease diagnostics, have also been associated with CIPN caused by agents such as oxaliplatin or taxanes [16–18]. Neurofilaments are structural neuronal proteins relevant for axonal integrity and conduction velocity [19, 20].

The aim of this study was to assess the prevalence of CIPN in adult cancer patients receiving systemic chemotherapy using a longitudinal design, standardized questionnaires, and ENG. We also analyzed associations with clinical variables such as age and sex and investigated whether CIPN is underdiagnosed in clinical oncological practice. Given the clinical need, we further explored the potential of serum NFL as a biomarker for early detection of CIPN as well as for continuous reassessments in daily outpatient oncological routine.

## Materials and methods

The ethical review committee of Charité–Universitätsmedizin Berlin approved this study (file reference: EA2/167/21). The study was conducted in accordance with the guidelines of Good Clinical Practice and the Declaration of Helsinki. Patients did not receive any financial compensation for participation.

### Patients

A total of 108 patients were included at the Department of Hematology, Oncology, and Tumour Immunology of Charité–Universitätsmedizin Berlin, Germany, between August 2021 and August 2023. Informed consent was obtained prior to the initiation of chemotherapy for the treatment of leukemia, lymphoma, or gastrointestinal malignancies. Inclusion criteria based on anamnestic information as well as pre-existing medical records were as follows: (1) ECOG (Eastern Cooperative Oncology Group) performance status 0 or 1 [34]; (2) a suspected life expectancy of at least 12 months; (3) legal capacity and a place of residence compatible with participation; (4) sufficient proficiency in German or English (speaking, reading, and understanding); (5) age between 18 and 85 years; (6) anamnestic no prior diagnosis of polyneuropathy; (7) a history of smoking ≤15 pack-years; (8) no current or previous COVID-19 infection or post-COVID syndrome; and (9) anamnestic no relevant otorhinolaryngological comorbidities. Study inclusion and follow-up examinations were conducted during regular medical appointments in the oncological outpatient clinic or hospital wards, meaning that no additional study visits or travel were required.

### Examination

Before the start of therapy, patients were screened for eligibility by reviewing their medical history, cancer characteristics (e.g., staging), and sociodemographic parameters predictive of successful study participation. Patients were assessed five times (T1–T5) over a period of 12–24 months. They were categorized into two groups for differentiated analysis: patients receiving temporary (with curative intention) chemotherapy and those undergoing continuous treatment. The first examination (T1) took place prior to the first chemotherapy cycle. In patients receiving temporary chemotherapy, the second visit (T2) occurred halfway through the planned treatment. The third assessment (T3) was conducted at the end of chemotherapy being around 6 months after T1. Follow-up visits were scheduled 3–6 months (T4) and 7–12 months (T5) after treatment completion at T3. In cases of relapse requiring a change or restart of chemotherapy, follow-up was discontinued. Patients undergoing continuous chemotherapy were assessed 3 months (T2), 6 months (T3), 9–12 months (T4), and 13–18 months (T5) after baseline. This means that evaluations at T4 and T5 were conducted at the same time in both groups. Evaluations during treatment were timed at the beginning of each chemotherapy cycle. All examinations were conducted either in the oncological outpatient clinic or in the hospital.

Examinations consisted of an extensive questionnaire especially focusing on symptoms of neuropathy, a clinical neurological evaluation, a nerve conduction measurement, and taking blood for examinations of NFL.

The study protocol included comprehensive questionnaires, comprising an expanded EORTC QLQ-C30 focusing on neuropathic symptoms, as well as a standardized questionnaire addressing smell and taste disorders, a clinical neurological examination, nerve conduction studies (NCS), and serum sampling for neurofilament light chain (NFL) measurement. At all dates, patients were asked about typical neurological positive and negative symptoms such as paresthesia, dysesthesia, numbness, burning pain, restless legs, and altered temperature perception. In addition, sociodemographic data were collected, including sex, age, height, weight, body mass index (BMI), and occupation. Quality of life was assessed using the EORTC QLQ-C30 questionnaire [21]. Further chemotherapy-related side effects such as reduced mobility, performance, appetite, or energy were documented.

After the questionnaire-based assessment, objective diagnostics followed. Peripheral neuropathy was evaluated at T1, T3, and T5 using NCS. Clinical neurological and electroneurographic examinations were performed on the less affected leg. Clinical neurological assessments included evaluation of muscle strength, reflexes, general sensation, temperature perception (thermoception), and vibration sense (pallesthesia). ENG measured the compound muscle action potential of the peroneal nerve and the sensory nerve action potential of the sural nerve. These electroneurographic measurements recorded amplitudes, nerve conduction velocities, and latencies. The results from NCS and clinical exams were summarized using the Total Neuropathy Score-reduced version (TNSr), a validated tool for quantifying nerve damage [22, 23]. Higher scores indicated more severe neuropathic involvement. Specifically, a TNSr of 0 indicated the absence of CIPN. Values between 1 and 10 corresponded to mild CIPN, while results between 11 and 19 indicated moderate CIPN. Higher values represented severe CIPN. NFL levels were analyzed from serum samples collected at each time point.

### Data analysis

Sociodemographic parameters were analyzed descriptively using medians, means, minimum and maximum values, standard deviations, and absolute and relative frequencies. Data analyses involved comparisons of multiple variables within various subgroups. These subgroups were stratified by factors such as age, sex, chemotherapy duration, neurotoxicity of the agents used, cytostatic drug classes, and documentation of a medical diagnosis of CIPN in clinical records. Normal distribution of data was evaluated using skewness and kurtosis. Frequency analyses were used to investigate longitudinal clinical and sociodemographic data across subgroups. Boxplots and line charts were used for graphical representation of key findings. Furthermore, non-parametric tests were employed. The Mann–Whitney U test was used to compare independent subgroups, while the Wilcoxon signed-rank test was applied for comparisons of dependent samples. Spearman correlation coefficients were calculated to determine associations and statistical significance between variables. Cross-tabulations were used to evaluate the sensitivity and specificity of different diagnostic approaches for CIPN. In addition, the potential of serum NFL as a biomarker for CIPN was assessed using receiver operating characteristic (ROC) curve analysis.

All statistical analyses were performed using SPSS Statistics version 27 (IBM Corp., Chicago, IL, USA). A significance threshold of *p* < 0.05 was applied.

## Results

### Sociodemographic and clinical characteristics

The most relevant characteristics of all included patients (*n* = 108), as well as those of the two most important subgroups, are presented in Table 1. These subgroups consist of patients receiving temporary chemotherapy (TC, *n* = 58) and patients undergoing continuous chemotherapy (CC, *n* = 50). In contrast to the CC group, patients in the TC group were predominantly treated with curative intent, primarily due to the high proportion of lymphomas in this subgroup. In comparison, patients receiving CC were generally older and more frequently diagnosed with gastrointestinal malignancies.

**Table 1:** Patients’ characteristics

Parameter	All patients	Patients receiving TC	Patients receiving CC
*n*	108	58	50
Mean age (SD) [years]	53.5 (17.6)	49.1 (19.1)	58.6 (14.1)
Female sex: *n* (%)	49.0 (45.4%)	22.0 (37.9%)	27.0 (54.0%)
Mean BMI (SD) [kg/m^2^]	24.7 (4.4)	24.2 (3.9)	25.2 (5.0)
ECOG score of 0: *n* (%)	101.0 (93.5%)	56.0 (96.6%)	45.0 (90.0%)
NT-treatment: *n* (%)	88 (81.5%)	48 (82.8%)	40 (80.0%)
Entity: *n* (%)			
Lymphoma	50 (46,3%)	46 (79.3%)	4 (8%)
Leukemia	13 (12%)	3 (5.2%)	10 (20.0%)
Gastrointestinal	45 (41,7%)	9 (15.5%)	36 (72%)
Cytostatic groups: *n* (%)			
Vinca alkaloids (%)	41 (38%)	36 (62%)	5 (10%)
Bortezomib (%)	2 (2%)	0 (0)	2 (4%)
Platinum (%)	34 (31%)	8 (14%)	26 (52%)
Taxane (%)	3 (3%)	0 (0%)	3 (6%)
Platinum + taxane (%)	3 (3%)	1 (2%)	2 (4%)
Other cytostatics (%)	22 (20%)	11 (19%)	11 (22%)
Missing information (%)	3 (3%)	2 (3%)	1 (2%)

*ECOG* Eastern Cooperative Oncology Group, *NT* neurotoxic, *TC* temporary chemotherapy, *CC* continuous chemotherapy

As a result of the differing distribution of oncological diagnoses between both groups, the cytostatic agents used also varied substantially between TC and CC.

Various reasons contributed to a progressive drop-out rate during this study (Table 2). A frequently reported reason for discontinuation—affecting 20 participants—was a change in treatment location due to the distance between their residence and the treatment center. In addition, 17 patients had to be excluded following disease relapse that required therapy modification, and 11 patients died as a result of their oncological diagnosis. Finally, 16 participants withdrew from the study because they felt too burdened to continue.

**Table 2:** Number of participating patients at each examination

Examination	All patients	Patients receiving TC	Patients receiving CC
T1	108	58	50
T2	89	50	39
T3	82	49	33
T4	63	39	24
T5	46	29	17

*TC* temporary chemotherapy, *CC* continuous chemotherapy

### Results of clinical neurological evaluations and electroneurography

**Fig. 1:**
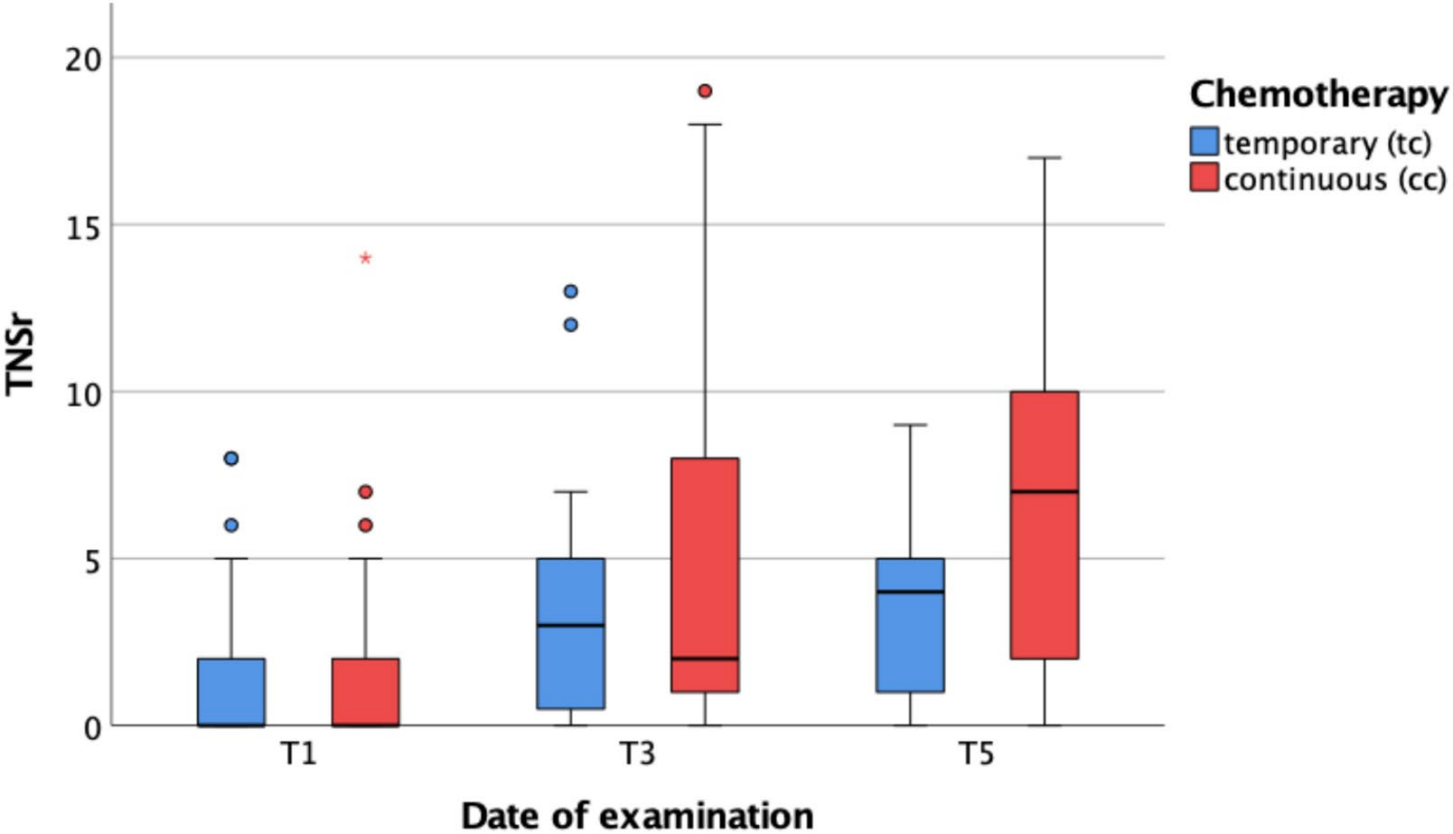
TNSr and intention of chemotherapy. *T1* before chemotherapy; *T3* at the end of temporary chemotherapy or 6 months into continuous chemotherapy; *T5* 6–12 months after temporary chemotherapy or 12–18 months into continuous chemotherapy

At T1, T3, and T5, electroneurography and clinical neurological evaluations were performed. The results of these assessments were summarized using the Total Neuropathy Score-reduced (TNSr) (Fig. 1). At baseline (T1), the median TNSr across the entire study population was 0. Between T1 and T3—during which all patients received chemotherapy—there was a statistically significant increase in TNSr in both groups (p < 0.001 for each). Between T3 and T5, TNSr values continued to rise significantly in both groups (TC: *p* = 0.027; CC: *p* = 0.004), with patients undergoing CC reaching higher TNSr values compared to those whose treatment ended at T3.

### Age and results of clinical neurological evaluations and electroneurography

At baseline (T1), 11% of patients aged 18–39 years showed TNSr values indicative of mild polyneuropathy. In comparison, this applied to 39% of patients aged 40–69 years and to 81% of those aged ≥ 70 years, suggesting a strong age-related increase in pre-existing neuropathic changes (Table 3). Across all age groups, TNSr values increased during chemotherapy. By T3, 63% of patients aged 18–39 years had developed TNSr scores corresponding to mild polyneuropathy. In both older age groups, the prevalence of polyneuropathy was even higher, and a greater proportion of patients exhibited more severe impairment (Table 4).

**Table 3:** Polyneuropathy and age at T1. CIPN: chemotherapy-induced polyneuropathy

Age group	No CIPN	Mild CIPN	Moderate CIPN	Severe CIPN
18–39	22	4	0	0
40–69	28	17	1	0
≥ 70	3	13	0	0

**Table 4:** Polyneuropathy and age at T3

Age group	No CIPN	Mild CIPN	Moderate CIPN	Severe CIPN
18–39	7	12	0	0
40–69	6	21	5	0
≥ 70	1	3	3	0

*CIPN* chemotherapy-induced polyneuropathy

### Sex and results of clinical neurological evaluations and electroneurography

**Fig. 2:**
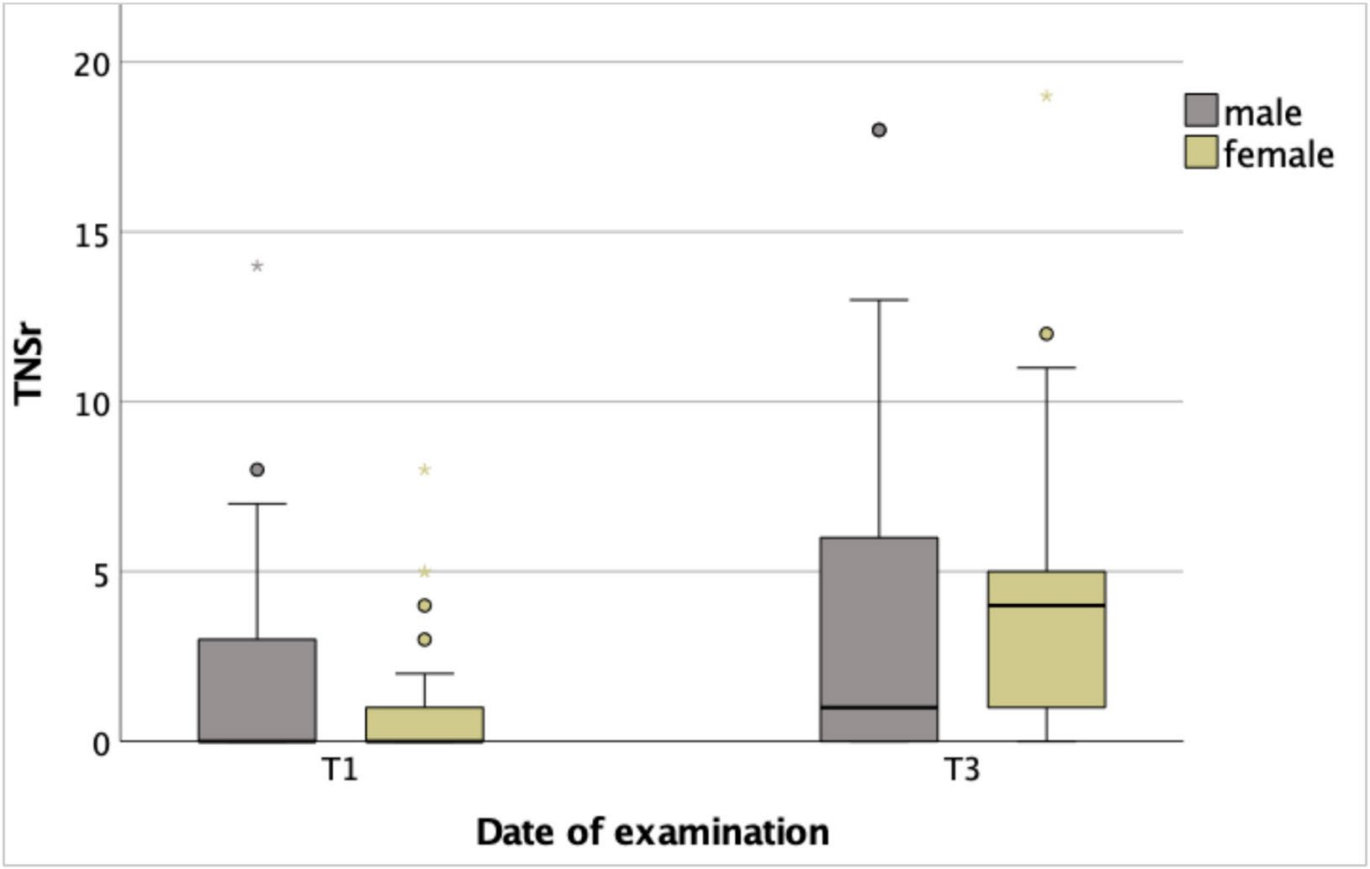
TNSr and sex during chemotherapy. *T1* before chemotherapy; *T3* at the end of temporary chemotherapy or 6 months into continuous chemotherapy

At baseline (T1), electroneurography and clinical neurological evaluations showed that 42% of male patients and 36% of female patients had elevated TNSr scores consistent with mild polyneuropathy. One patient exhibited a TNSr indicating moderate polyneuropathy at baseline. All remaining patients had a TNSr of 0 prior to chemotherapy, suggesting no detectable neuronal damage. Following chemotherapy, TNSr values increased in both sexes; however, female patients exhibited higher scores overall (Fig. 2).

### Neurotoxicity and results of clinical neurological evaluations and electroneurography

**Fig. 3:**
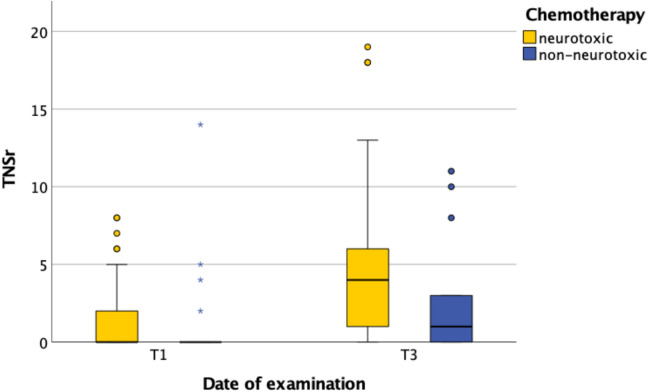
TNSr and neurotoxicity during chemotherapy. *T1* before chemotherapy; *T3* at the end of temporary chemotherapy or 6 months into continuous chemotherapy

When dividing patients into two groups based on neurotoxic—including taxanes, vinca alkaloids, bortezomib, or oxaliplatin—versus non-neurotoxic—including bendamustine, irinotecan, or fluorouracil—chemotherapies, we observed a differing increase in TNSr from T1 to T3. Patients receiving neurotoxic chemotherapy protocols showed a higher, though not statistically significant, average TNSr at T3 (*p* = 0.138). In addition, patients treated with neurotoxic agents reached higher peak TNSr values (Fig. 3). Among the neurotoxic therapies at T3, patients receiving taxanes demonstrated the greatest increase in TNSr, with an average of 14.5 points (*n* = 4), compared to 4.0 points with vinca alkaloids (*n* = 24) and 2.5 points with oxaliplatin (*n* = 12).

### CIPN diagnosis by TNSr vs. clinical diagnosis by treating oncologists

At T3, 18 patients had been diagnosed with CIPN, according to medical records of treating oncologists. In comparison, based on electroneurography and neurological assessments reflected by the TNSr, 42 patients met the criteria for a CIPN diagnosis. This corresponds to a sensitivity of 42.9% with a confidence interval from 27.3 to 58.5%. Notably, all patients clinically diagnosed with CIPN also showed pathological findings in electroneurography, resulting in a specificity and positive predictive value of 100% (Table 5).

**Table 5:** Comparison of CIPN diagnosis by TNSr and clinical assessment at T3. CIPN: chemotherapy-induced polyneuropathy

	Clinical diagnosis of CIPN		Total
	NO CIPN	CIPN	
TNSr-based diagnosis of CIPN			
No CIPN	14	0	14
CIPN	24	18	42
Total	38	18	56

At T5, the comparison between clinical documentation and electroneurographic results revealed a pattern similar to that observed at T3. According to the TNSr, 19 patients met the criteria for CIPN, while only 9 of these had been clinically diagnosed with CIPN by their oncologists. This corresponds to a sensitivity of 47.4% with a confidence interval from 22.6 to 72.1%. Interestingly, one patient had been clinically diagnosed with CIPN despite showing no abnormalities in the TNSr. In total, specificity in this small group reached 75% and the positive predictive value reached 90% (Table 6).

**Table 6:** Comparison of CIPN diagnosis by TNSr and clinical assessment at T5.CIPN: chemotherapy-induced polyneuropathy

	Clinical diagnosis of CIPN		Total
	NO CIPN	CIPN	
TNSr-based diagnosis of CIPN			
No CIPN	3	1	4
CIPN	10	9	19
Total	13	10	23

### Results of NFL

**Fig. 4:**
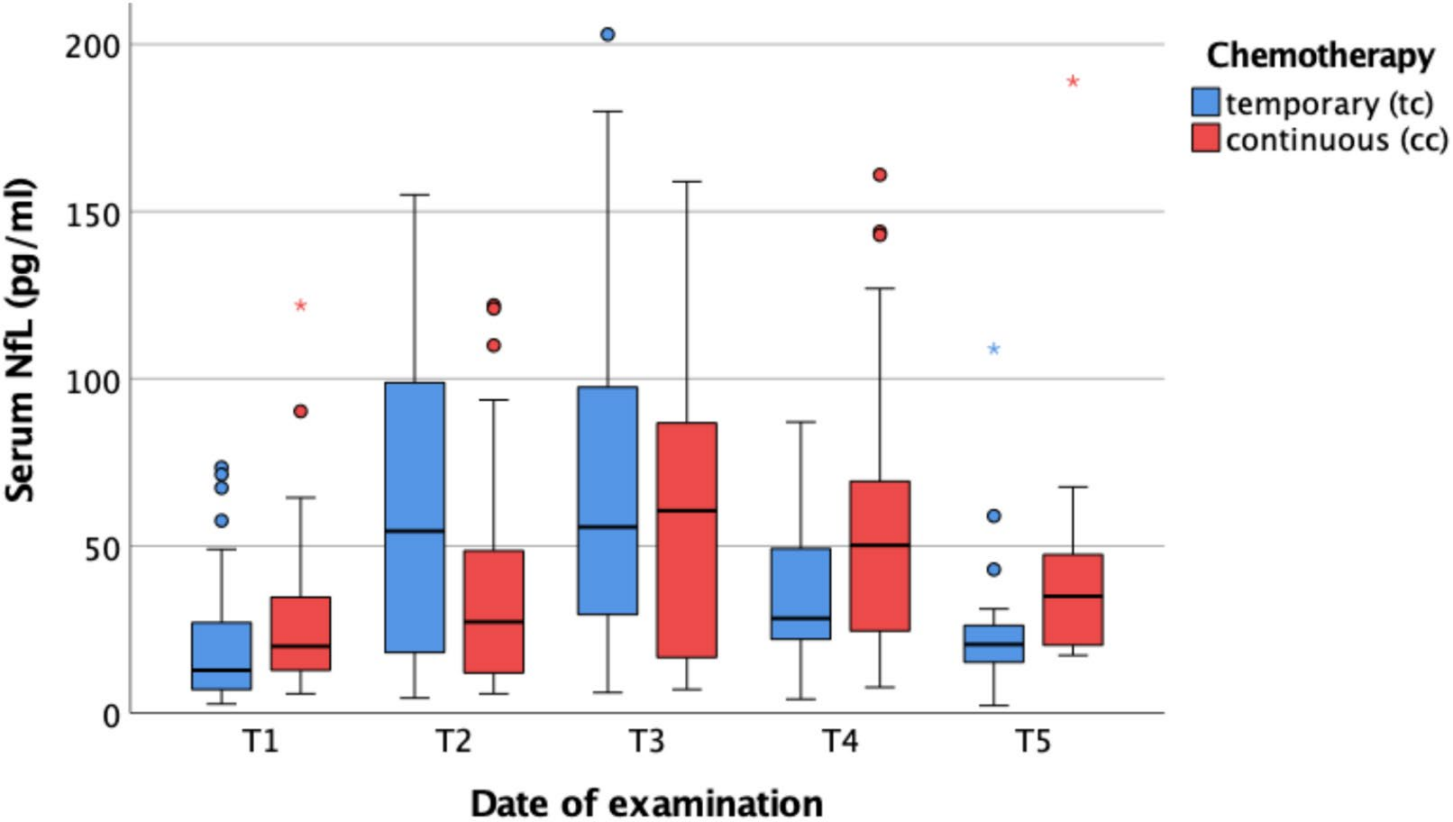
NFL and intention of chemotherapy. *T1* before chemotherapy; *T2* during chemotherapy; *T3* at the end of temporary chemotherapy or 6 months into continuous chemotherapy; *T4* 3–6 months after temporary chemotherapy or 9–12 months into continuous chemotherapy; *T5* 6–12 months after temporary chemotherapy or 12–18 months into continuous chemotherapy

Prior to the beginning of chemotherapy, the median serum NFL level in 102 patients without CNS involvement was 15.35 pg/ml. Patients were analyzed in two subgroups: those receiving TC and those receiving CC. During chemotherapy, NFL levels increased significantly in both groups from T1 to T3 (*p* < 0.001). The median NFL level at T3 was 55.7 pg/ml in the TC group and 60.55 pg/ml in the CC group. After cessation of chemotherapy, NFL levels in the TC group decreased significantly from T3 to T5 (*p* < 0.001). In contrast, the CC group showed only a slight, non-significant decrease in NFL levels over the same period. As a result, at T5, NFL levels in the CC group remained significantly higher compared to the TC group (Fig. 4). When interpreting the post-T3 development of NFL values in the CC group, it is important to consider that treatment protocols are often modified and dose-reduced after approximately 6 months. These so-called maintenance therapies, following initial induction treatments and used to manage side effects, are particularly frequently adjusted with respect to the dosages of neurotoxic chemotherapeutics. Moreover, for the interpretation of results, age-adjusted standard values of NfL should be considered: < 7.4 pg/ml for individuals under 20 years, < 9.9 pg/ml for those under 30 years, < 13.1 pg/ml for those under 40 years, < 17.5 pg/ml for those under 50 years, < 23.3 pg/ml for those under 60 years, < 30.9 pg/ml for those under 70 years, and < 41.3 pg/ml for those under 80 years.

In contrast, patients with CNS-affecting diagnoses (lymphoma or ALL) showed strongly elevated NFL levels at baseline with a median exceeding 300 pg/ml. Each patient showed good treatment response, which led to rapidly declining and later normalized NFL levels during subsequent chemotherapy.

### Neurotoxicity and NFL

**Fig. 5:**
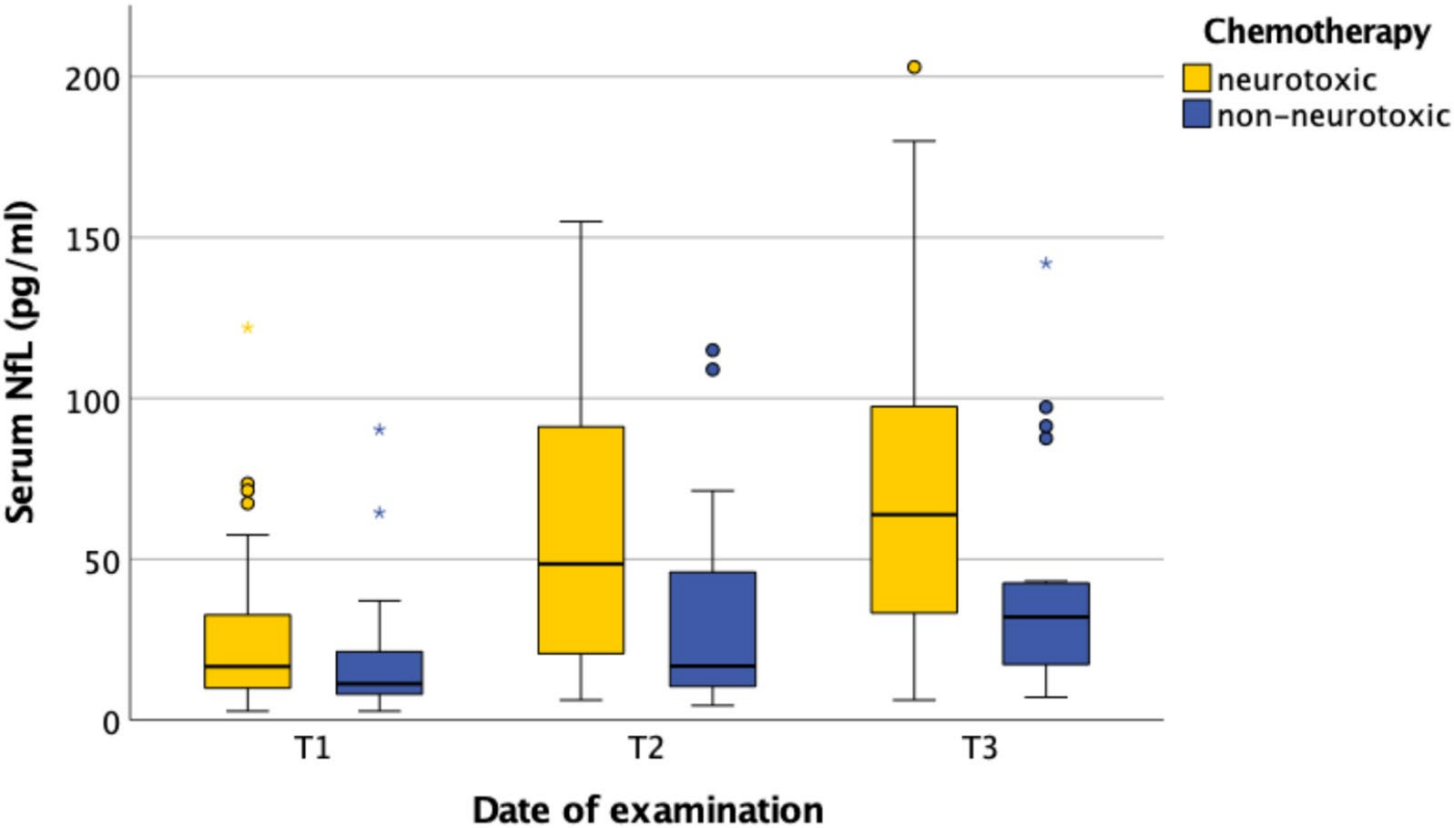
NFL and neurotoxicity during chemotherapy. *T1* before chemotherapy; *T2* during chemotherapy; *T3* at the end of temporary chemotherapy or 6 months into continuous chemotherapy; *T4* 3–6 months after temporary chemotherapy or 9–12 months into continuous chemotherapy; *T5* 6–12 months after temporary chemotherapy or 12–18 months into continuous chemotherapy

When comparing specific cytotoxic agents, chemotherapy protocols containing known neurotoxic drugs—such as taxanes, oxaliplatin, and vinca alkaloids—led to significantly greater increases in NFL levels than protocols without neurotoxic—such as bendamustine, irinotecan, and fluorouracil—agents. The difference in NFL levels between neurotoxic and non-neurotoxic protocols reached statistical significance both between T1 and T2 (*p* = 0.007) and between T1 and T3 (*p* = 0.016) (Fig. 5).

**Fig. 6:**
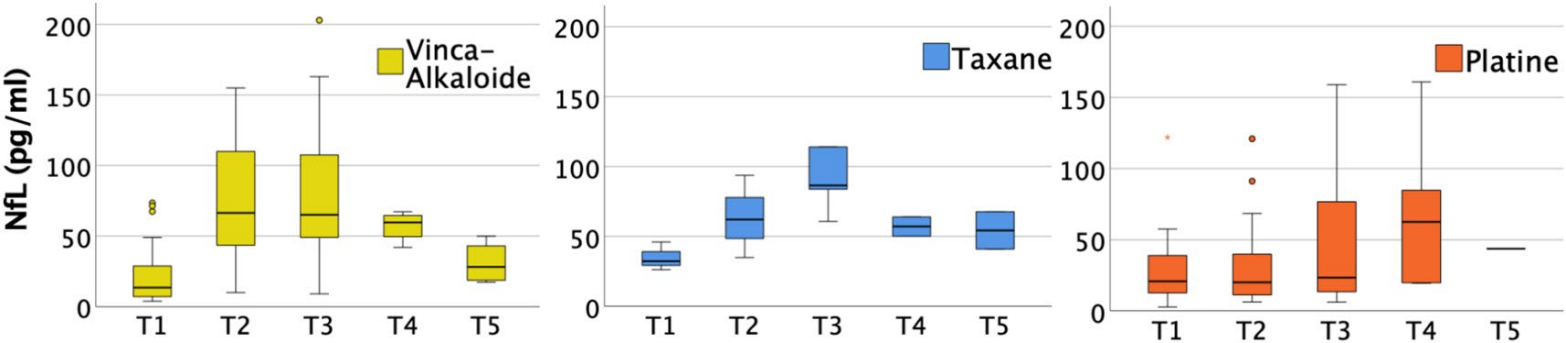
NFL and different neurotoxic drugs. *T1* before chemotherapy; *T2* during chemotherapy; *T3* at the end of temporary chemotherapy or 6 months into continuous chemotherapy; *T4* 3–6 months after temporary chemotherapy or 9–12 months into continuous chemotherapy; *T5* 6–12 months after temporary chemotherapy or 12–18 months into continuous chemotherapy

Further analysis of subgroups receiving different neurotoxic agents—such as vinca alkaloids, taxanes, and oxaliplatin—revealed a significant increase in NFL levels across all subgroups (Fig. 6). Patients treated with vinca alkaloids showed a more rapid rise in NFL levels compared to those receiving oxaliplatin. After T3, NFL levels decreased in patients treated with vinca alkaloids. Interpretation of these findings must take into account that most patients receiving vinca alkaloids were part of the TC group, whereas patients treated with oxaliplatin primarily belonged to the CC group.

In addition, a small number of patients receiving taxanes, as well as two patients receiving bortezomib, were included. At baseline, NFL levels in the patients receiving bortezomib measured 16 and 36 pg/ml, respectively. By T3, both showed a marked increase, reaching 83 and 86 pg/ml. Patients receiving taxanes had a median NFL of 32.3 pg/ml at baseline, which increased to a median of 86.5 pg/ml by T3. At T5, only two patients were still receiving continuous chemotherapy with taxanes; their NFL levels decreased to 41 and 68 pg/ml, respectively.

### Results of subjective evaluations of CIPN

**Fig. 7:**
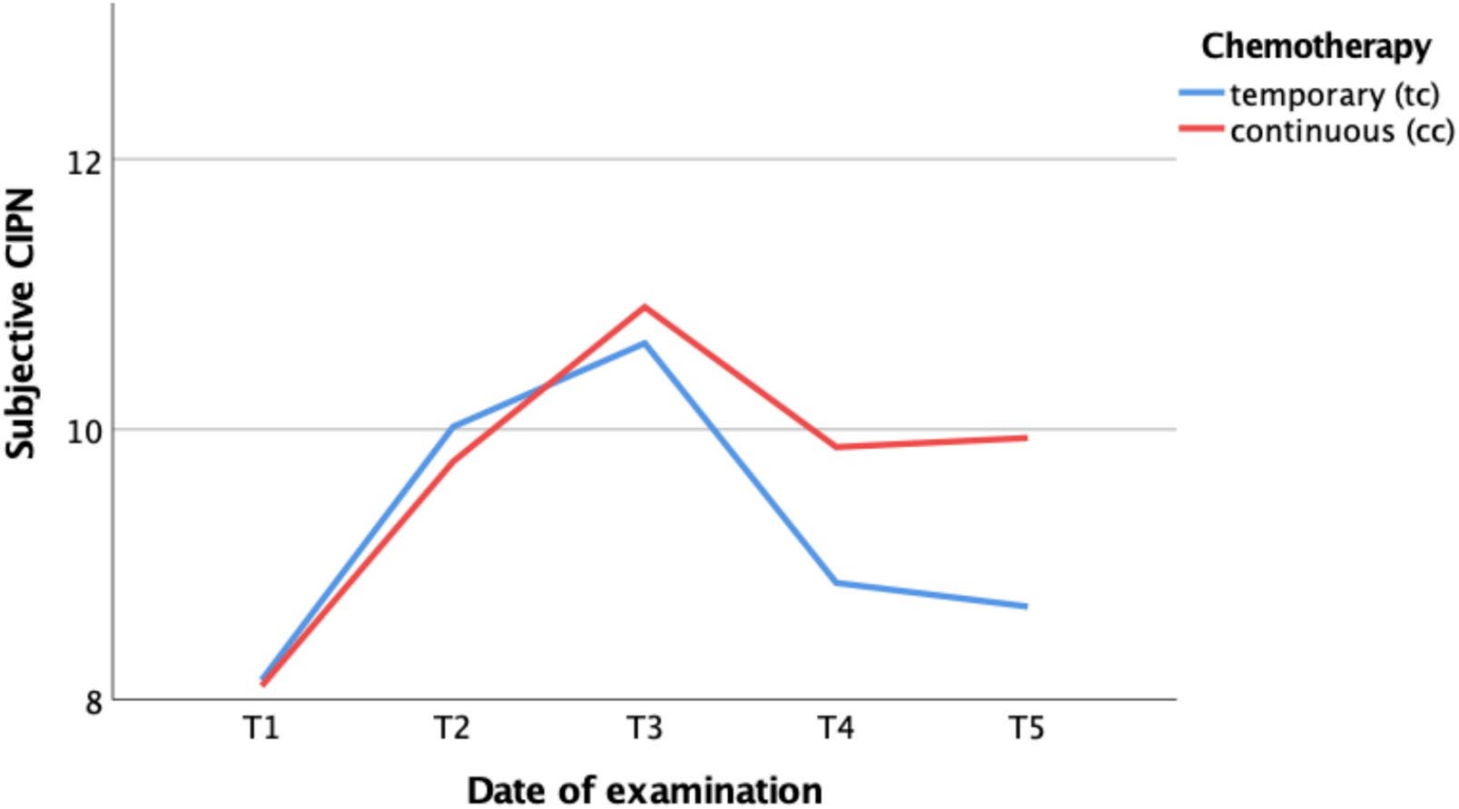
Severity of self-reported CIPN. *T1* before chemotherapy; *T2* during chemotherapy; *T3* at the end of temporary chemotherapy or 6 months into continuous chemotherapy; *T4* 3–6 months after temporary chemotherapy or 9–12 months into continuous chemotherapy; *T5* 6–12 months after temporary chemotherapy or 12–18 months into continuous chemotherapy

In addition to the analysis of objective measures, patient-reported outcomes—such as quality of life and positive as well as negative symptoms of polyneuropathy—also revealed distinct developments during chemotherapy. Both the frequency and severity of self-reported CIPN increased. At T2, nearly half of all patients reported neuropathic symptoms such as numbness in the extremities or paresthesia. At T3, 63% of patients reported symptoms consistent with CIPN. Following the completion of cytotoxic therapy, the prevalence of these symptoms declined: by T5, only one-third of patients who had received temporary chemotherapy still reported CIPN symptoms. In contrast, patients undergoing CC did not show a relevant reduction in the frequency of self-reported CIPN at T5. Furthermore, not only the frequency but also the severity of CIPN symptoms decreased after cessation of chemotherapy. The reported severity differed significantly between patients receiving temporary and continuous chemotherapy (*p* = 0.016) (Fig. 7). Again, interpretation of reduced CIPN symptoms need to consider frequent reduction or modification of dosage during CC. Especially after T3, in everyday clinical practice, chemotherapy often shifts from induction to maintenance therapy.

## Discussion

Our data reinforce the well-established association between chemotherapy in adult cancer patients and the frequently observed side effect of CIPN [6]. Primarily based on clinical neurological assessments as well as on electroneurography results at T3, 75% of patients showed an increased TNSr indicative of CIPN. Nonetheless, our findings confirm the substantial clinical variability of CIPN in terms of both frequency and severity. Many factors influence the clinical manifestation of CIPN. On the one hand, the occurrence of CIPN depends on therapy-related risk factors. Analysis of TNSr across subgroups demonstrated a significant increase in both frequency and severity of CIPN associated with the use of cytotoxic drugs and longer chemotherapy duration [7, 24]. At T3, patients receiving neurotoxic chemotherapy showed greater TNSr increase (*p* = 0.138) and higher total TNSr. Furthermore, different neurotoxic subgroups—oxaliplatin, vinca alkaloids, and taxanes—displayed varying TNSr profiles. On the other hand, patient-related factors also influence the likelihood of developing CIPN. Relevant risk factors highlighted by our results include older age and pre-existing polyneuropathy [25]. In contrast, no sex-related differences were observed. In summary, CIPN occurrence depends on multiple patient- and therapy-related parameters.

This high variability poses a significant diagnostic challenge, particularly in routine outpatient oncology, due to limited time and the lack of advanced diagnostic capabilities, as also emphasized by our data [13]. Comparison of clinical diagnoses documented in medical records of treating oncologists with results of clinical neurological and electroneurographic assessments presented by TNSr clearly showed the extent of underdiagnosed CIPN, as less than half of all CIPN cases were identified by oncological clinicians in daily routine. Notably, undetected cases included not only mild but also some moderate polyneuropathies. Overall, clinical diagnosis shows low sensitivity but high specificity.

Due to time and resource constraints in everyday oncological practice, CIPN diagnosis currently relies mainly on patient reports and often superficial clinical neurological examinations. Structured questionnaires could offer a standardized approach to collecting patient information, but these tools are often too time-consuming for routine use [26, 27]. Nevertheless, our assessments of patient-reported symptoms reveal that over 60% of patients reported CIPN symptoms at T3. In contrast, only 32% of patients had a documented diagnosis of CIPN at that time. When considering the even more pronounced findings from clinical neurological and electroneurographic examinations, this large discrepancy between patient reports and clinical documentation underscores the current inadequacy of CIPN diagnosis in clinical practice. Limited consultation time per patient is likely a key factor contributing to this extent of underdiagnosis. These findings highlight the urgent need to improve current CIPN diagnostic procedures in routine care.

Given the current circumstances, CIPN diagnosis and continuous reassessments in outpatient oncological daily routine needs to be improved quickly without additional staff. A practical solution could be the use of a biomarker indicating acute nerve damage. Given its specific expression in neurons and its established diagnostic value in other neurological diseases, neurofilament light chain (NFL) may serve as a useful biomarker for CIPN and warrants further evaluation [28, 29]. It should be noted, however, that many neurological studies have measured NFL in cerebrospinal fluid (CSF) [28, 29]. Nonetheless, smaller amounts of NFL are also detectable in serum and correlate with CSF levels [28, 29]. Nevertheless, smaller quantities of NFL also appear in serum correlating with values in liquor [30]. Considering time constraints and the preference for less invasive diagnostic tools, CSF assessments are impractical for routine CIPN diagnosis, except in special cases. Based on similar reasoning, previous studies have demonstrated increased serum NFL levels in patients treated with oxaliplatin [17], paclitaxel [18], and bortezomib [31]. Our results support these findings, showing significantly higher levels in serum NFL in patients receiving neurotoxic chemotherapies compared to those receiving non-neurotoxic regimens at both T2 (*p* = 0.007) and T3 (*p* = 0.016). Within neurotoxic subgroups, all patients exhibited relevant NFL increases; however, patients treated with oxaliplatin showed a slower rise compared to those receiving taxanes, vinca alkaloids, or bortezomib. This observation aligns with previous electrophysiological data. It should be noted that subgroup sizes were uneven as less patients receiving taxanes and bortezomib were assessed. The differences in NFL increase and variations in TNSr among neurotoxic subgroups at T3 may reflect distinct pathophysiological or pharmacological mechanisms, presenting an interesting approach for further research. Also, after cessation of chemotherapy, NFL levels decreased while TNSr continued to increase between T3 and T5. This proves that electroneurography is more effective in detecting and quantifying persistent neurological damage, as electroneurographic changes occur later than the initial neuronal injury reflected by NFL elevations. The early rise of NFL following neurotoxic drug exposure and its decrease after therapy cessation support the hypothesis that NFL could be a suitable marker for acute, but not chronic, neuronal damage. Overall, serum NFL shows potential as a useful biomarker for CIPN diagnosis and continuous reassessments in daily outpatient oncological routine. However, further studies with larger patient cohorts for each specific neurotoxic drug are needed to confirm its utility in routine clinical practice, alongside established diagnostic standards and considering its limitations.

Finally, the observation of markedly elevated NFL levels in patients with CNS involvement appears highly relevant. This warrants further investigation regarding the potential role of NFL as an unspecific tumor marker detecting CNS involvement. In addition, the decline in NFL levels in response to effective therapy highlights its potential as a marker for monitoring disease progression.

## Conclusion

This study builds on existing knowledge of chemotherapy-induced peripheral neuropathy (CIPN) in cancer patients and highlights the challenges in managing this frequent side effect in daily oncological routine. Routine use of electroneurography and neurological examinations revealed a high prevalence of CIPN (75–83%) and considerable clinical variability. Notably, elevated TNSr values persisted even after the cessation of systemic therapy, indicating that CIPN is, at least in part, a long-lasting side effect lasting for more than 1 year. Furthermore, we identified both patient-related and therapy-associated risk factors for CIPN. These included advanced age and pre-existing polyneuropathy as well as the use of neurotoxic agents and longer treatment duration.

However, our findings also demonstrate that clinical diagnosis of CIPN in daily oncological routine—primarily based on patients’ subjective reports due to limited time and the lack of advanced diagnostic capabilities—detects less than half of all cases. The use of objective biomarkers such as neurofilament light chain (NFL) in serum could help close this gap. In our study, patients receiving neurotoxic chemotherapies showed a significant increase in serum NFL during treatment. This pattern was observed across all neurotoxic subgroups, including vinca alkaloids, oxaliplatin, taxanes, and bortezomib. It should be noted that the neurotoxic subgroups varied in size. Interestingly, oxaliplatin was associated with a slower NFL increase. After chemotherapy, serum NFL levels declined over time, supporting the hypothesis that rising NFL levels may specifically indicate acute neurotoxic damage. In contrast, extensive clinical neurological examinations and electroneurography appear suitable for detection of chronic, structural neuronal damage. Together, these findings suggest that NFL may serve as a biomarker for acute neuronal injury, while TNSr remains important for assessing persistent neurological impairment and for validating suspected diagnoses. However, further studies with larger patient cohorts in each neurotoxic subgroup are needed to validate these findings. Lastly, the small subgroup of patients with CNS-involving malignancies exhibited initially elevated NFL levels, which declined in response to effective therapy. This suggests a potential role for NFL not only as a diagnostic but also as a prognostic marker in such cases.

In summary, the current detection rate of CIPN in clinical practice remains unsatisfactory. Supplementing standard diagnostic approaches with serum NFL measurement may improve early detection, reassessments, and management of CIPN. In addition, the potential of NFL as a tumor or progression marker in patients with CNS involvement should be further explored.

## Data Availability

On request, the raw data of this article pointing up our conclusions can be made available by the authors.
